# OLTW-TEC: online learning with sliding windows for text classifier ensembles

**DOI:** 10.3389/frai.2024.1401126

**Published:** 2024-09-11

**Authors:** Khrystyna Lipianina-Honcharenko, Yevgeniy Bodyanskiy, Nataliia Kustra, Andrii Ivasechkо

**Affiliations:** ^1^Department of Information Computer Systems and Control, West Ukrainian National University, Ternopil, Ukraine; ^2^Department of Artificial Intelligence, Kharkiv National University of Radioelectronics, Kharkiv, Ukraine; ^3^Department of Publishing Information Technologies, Lviv Polytechnic National University, Lviv, Ukraine

**Keywords:** disinformation, fake news, online learning, classifier ensembles, machine learning

## Abstract

In the digital age, rapid dissemination of information has elevated the challenge of distinguishing between authentic news and disinformation. This challenge is particularly acute in regions experiencing geopolitical tensions, where information plays a pivotal role in shaping public perception and policy. The prevalence of disinformation in the Ukrainian-language information space, intensified by the hybrid war with russia, necessitates the development of sophisticated tools for its detection and mitigation. Our study introduces the “Online Learning with Sliding Windows for Text Classifier Ensembles” (OLTW-TEC) method, designed to address this urgent need. This research aims to develop and validate an advanced machine learning method capable of dynamically adapting to evolving disinformation tactics. The focus is on creating a highly accurate, flexible, and efficient system for detecting disinformation in Ukrainian-language texts. The OLTW-TEC method leverages an ensemble of classifiers combined with a sliding window technique to continuously update the model with the most recent data, enhancing its adaptability and accuracy over time. A unique dataset comprising both authentic and fake news items was used to evaluate the method’s performance. Advanced metrics, including precision, recall, and F1-score, facilitated a comprehensive analysis of its effectiveness. The OLTW-TEC method demonstrated exceptional performance, achieving a classification accuracy of 93%. The integration of the sliding window technique with a classifier ensemble significantly contributed to the system’s ability to accurately identify disinformation, making it a robust tool in the ongoing battle against fake news in the Ukrainian context. The application of the OLTW-TEC method highlights its potential as a versatile and effective solution for disinformation detection. Its adaptability to the specifics of the Ukrainian language and the dynamic nature of information warfare offers valuable insights into the development of similar tools for other languages and regions. OLTW-TEC represents a significant advancement in the detection of disinformation within the Ukrainian-language information space. Its development and successful implementation underscore the importance of innovative machine learning techniques in combating fake news, paving the way for further research and application in the field of digital information integrity.

## 1 Introduction

During the russian-Ukrainian war, the importance of detecting fake news gains particular relevance, as disinformation can have serious consequences. The research by Tao and Peng (2023) and Mainych et al. (2023) emphasizes how social media, particularly Twitter and Weibo, play a key role in shaping public opinion during the conflict. The analysis shows that at the beginning of the war, there was a significant spike in activity, which later decreased but continued to draw attention to Ukraine as a victim of aggression. The importance of distinguishing be-tween real and fake news becomes even more critical, considering that social media is often used as the primary source of information. Thus, there is an increasing need for the development of effective methods for detecting and analyzing fake news to ensure the accuracy and safety of information. In this context, the development of effective methods for disinformation detection is critically important. This study offers an innovative approach that leverages the advantages of classifier ensembles combined with online learning methods. This approach allows not only for accurate identification of fake news but also for adaptation to the constantly changing information environment, ensuring the relevance and effectiveness of disinformation detection in real-time.

In addition to the growing relevance of disinformation detection during the russian-Ukrainian war, the broader research context highlights a gap in existing methodologies for addressing disinformation in non-English languages, particularly Ukrainian. While there has been substantial progress in the development of disinformation detection tools for English and other widely spoken languages, the unique linguistic and cultural characteristics of Ukrainian content require specialized approaches. This study seeks to fill this gap by developing a method that not only addresses the challenges of disinformation detection in the Ukrainian-language information space but also adapts to the dynamic and non-stationary nature of online news. By focusing on these specific needs, this research contributes to the global effort of combating disinformation and ensuring information integrity in regions severely affected by conflict. Furthermore, the study emphasizes the importance of a transparent data collection process, which is critical for the reproducibility of results and the broader applicability of the OLTW-TEC method in diverse linguistic contexts.

This research presents the development and analysis of the innovative method Online Learning with Sliding Windows for Text Classifier Ensembles (OLTW-TEC), which combines the advantages of adaptive online learning with a dynamic sliding window for text classifier ensembles. The goal is to increase the accuracy and adaptability in identifying fake news, particularly in the Ukrainian-language information space. This work highlights the importance of developing tools capable of adequately working with the unique linguistic and cultural features of specific language groups, as well as the necessity of ensuring the high responsiveness of systems in response to rapid changes in the information flow.

## 2 Related work

This comprehensive survey explores the landscape of fake news detection research, highlighting the diverse methodologies and technological innovations developed by scholars to tackle the ever-evolving challenge of identifying disinformation across various languages, platforms, and contexts.

Hamed et al. (2023) and Kondamudia et al. (2023) conduct review studies, analyzing various approaches to detecting fake news. Hamed and co-authors focus on challenges associated with datasets, feature representation, and data integration, while Kondamudia and co-authors examine attributes, features, and methods of detecting fake news in social networks, including linguistic and semantic analysis. In the studies by Hu et al. (2022) and Phan et al. (2023), the focus is on the use of deep learning and graph neural networks for detecting fake news. Hu and co-authors consider various deep learning approaches, including supervised, weakly supervised, and unsupervised learning, and analyze their effectiveness based on different datasets. Phan and co-authors focus on the use of graph neural networks, pointing out their potential and challenges related to data standardization and the development of specialized hardware.

The study by Das and Tsb (2023) highlights the importance of multi-contextual learning in the study of disinformation, considering various contexts such as content, emotions, users, and others. The authors offer a comprehensive view of this issue, including challenges associated with the multimodality of content and the scarcity of labeled data. Ruffo et al. (2023) also emphasize the necessity of an interdisciplinary approach, including network and language analysis, to understand the dynamics of the spread of fake news and its impact.

In the article by Baker et al. (2023), the way people express their feelings on Twitter during the conflict between Russia and Ukraine is investigated. The study has two objectives: to collect unique data and to use machine learning (ML) to classify tweets depending on their impact on people’s emotions. The first goal was to identify the most relevant hashtags related to the conflict to find a dataset. The second goal was to use several well-known ML models to group tweets. Experimental results showed that most ML classifiers have higher accuracy on a balanced dataset. However, the results of experiments using data balancing strategies do not necessarily indicate that all classes will perform better. Therefore, it’s important to emphasize the im-portance of comparing and contrasting data balancing strategies used in SA and ML research, including more classifiers and a broader range of use cases.

In the article, the authors Chang et al. (2024) introduce a new approach to detecting fake news using deep learning, employing natural language processing (NLP) techniques for encoding nodes with the context of news and users. They implement three graph convolutional networks to extract informative features from the news dissemination network and aggregate both internal and external user information. The methodology includes a global attention mechanism with memory for learning the structural homogeneity of news dissemination networks and a module for aggregating partial key information. Experimental results demonstrate the effectiveness of the proposed approach in detecting fake news, achieving high accuracy and F1-score metrics on real datasets. For instance, the GCN-GANM model showed high accuracy (Acc.: 0.9825, F1: 0.9825) on the Gossipcop dataset and (Acc.: 0.9804, F1: 0.9805) on the Weibo dataset, outperforming other methods. This work proposes a new direction in fake news detection research, combining global and partial information.

In the article, the authors Peng et al. (2024) developed a new method for detecting fake news that considers contextual semantic representation for analyzing multimodal data in social networks. This method, named CSFND (Contextual Semantic representation learning for multimodal Fake News Detection), includes an unsupervised learning phase of context to obtain local contextual features of news, which are then combined with global semantic features for learning the news’ contextual semantic representation. Experiments on two real multimodal datasets showed that CSFND significantly outperforms 10 state-of-the-art fake news detection methods, improving the average accuracy by 2.5% compared to the best baseline methods.

In the article by Ahammad (2024), the spread of fake news about COVID-19, which has become a critical issue, is investigated. The study aims to gain insights into the types of disinformation being spread and to develop a deep analytical approach for analyzing fake news about COVID-19. It combines sentiment analysis and topic modeling to improve the accuracy of extracting themes from a large volume of unstructured texts, considering the sentiment of words. A dataset containing 10,254 news headlines from various sources was collected and prepared, and rule-based SA was applied to tag the dataset with three sentiment tags. Among the evaluated TM models, Latent Dirichlet Allocation showed the highest coherence score of 0.66 for 20 clustered themes with negative sentiment and 0.573 for 18 clustered positive fake news themes, outperforming Non-negative Matrix Factorization (coherence: 0.43) and Latent Semantic Analysis (coherence: 0.40). The identified themes highlight that disinformation predominantly revolves around COVID vaccines, crimes, quarantine, medicine, and political and social aspects. This study provides insights into the impact of fake news about COVID-19, offers a valuable method for detecting and analyzing disinformation, and underscores the importance of understanding the patterns and themes of fake news to protect public health and promote scientific accuracy.

In the article by Qu et al. (2023), a new model for detecting fake news on social networks was developed, based on quantum multimodal fusion (QMFND). The QMFND model integrates extracted image and text features, which go through a proposed quantum convolutional neural network (QCNN) to obtain discriminative results. Testing QMFND on two social media datasets, Gossip and Politifact, showed that its performance is equal to or even exceeds that of classic models. Furthermore, the impact of various parameters was explored. QCNN proved to be not only highly expressive and capable of entanglement but also resistant to quantum noise.

In another article, Farhangian et al. (2024) conducted a detailed study in the field of fake news detection, proposing an updated taxonomy for this domain based on several criteria: types of used features, perspectives on fake news detection, methods of feature representation, and approaches to classification. The authors conducted a wide-ranging empirical study, evaluating various feature representation techniques and classification approaches based on accuracy and computational costs. Experimental results showed that optimal feature extraction techniques depend on the characteristics of the dataset. Transformer-based models consistently demonstrated higher performance. Moreover, using transformer models as feature extraction methods, rather than merely fine-tuning the network for post-activity, improves overall performance. Through careful error analysis, it was discovered that a combination of feature representation methods and classification algorithms, including classical ones, offer complementary aspects and should be considered to achieve better overall performance while maintaining relatively low computational costs.

In the article by Fang et al. (2024), a new approach to the early detection of fake news through the perception of the news semantic environment (NSEP) is introduced. NSEP utilizes graph convolutional networks to detect semantic inconsistencies between the content of news and external posts, as well as a microsemantic detection module with multi-head and sparse attention to identify semantic contradictions. Experiments on real Chinese and English datasets showed that NSEP achieves an accuracy of up to 86.8% on Chinese datasets, which is 14.1% higher than other methods. This confirms the effectiveness of detecting fake news through the analysis of micro- and macrosemantic environments.

In the article by Soga et al. (2023), the problem of detecting fake news on social networks is examined. The authors propose a new approach that considers the similarity of user opinions by analyzing their positions regarding news articles and interactions in posts. Using a network of graph transformers, the method simultaneously extracts global structural information and interactions of similar positions. The method was evaluated on specially collected data from Twitter and the FibVID dataset, demonstrating significant improvement compared to traditional methods, including state-of-the-art approaches.

In the article by Yang et al. (2024), the problem of detecting fake news in multimodal data is examined. The authors highlight the challenges related to analyzing the relationships between regions of images and fragments of text, as well as the necessity for a deeper analysis of hierarchical text semantics. To address these issues, a Multimodal Relation Attention Network (MRAN) is proposed, which includes several processing stages. Initially, a multi-level encoding network is used to extract semantic features of the text, along with VGG19 for extracting visual characteristics. Then, an attention network is utilized to compute the similarity between segments of information within and across modalities. Finally, the obtained features are fed into a fake news detector. Experiments on three datasets demonstrated the effectiveness of MRAN, underscoring its strong performance in detecting fake news.

In the article by Raja et al. (2024), the problem of detecting fake news in Dravidian languages, which are low-resourced, is discussed. The authors propose a hybrid deep learning model that integrates Enhanced Temporal Convolutional Neural Networks (DTCN), Bidirectional Long Short-Term Memory (BiLSTM), and Contextualized Attention Mechanism (CAM). DTCN is used for capturing temporal dependencies, BiLSTM for effectively capturing long-term dependencies, and CAM for emphasizing important information and reducing the impact of irrelevant content. The model also employs an adaptive cyclic learning rate with an early stopping mechanism to improve model convergence. The results show that the proposed model outperforms existing methods and achieves a high average accuracy of 93.97% on the Dravidian_Fake dataset in four Dravidian languages.

In the article by Luvembe et al. (2024), the issue of detecting fake news in a multimodal context is explored. The authors point out the shortcomings of existing methods that integrate cross-modal features without considering uncorrelated semantic representations, which can introduce noise into the multimodal characteristics. This lowers the accuracy of models, as it is crucial to account for the subtle differences between text and images in identifying fake news. To overcome these challenges, the CAF-ODNN model is proposed, which utilizes complementary attention and an optimized deep neural network to detect subtle cross-modal connections. The model includes generating captions for images for semantic representation, bidirectional complementary attention between modalities, and an alignment and normalization component for calibrating fused representations. The Optimized Deep Neural Network (ODNN) is used to enhance feature extraction. The model outperforms similar approaches on standard metrics across four real-world datasets, highlighting the importance of complementary attention and optimization in detecting fake news.

The article by Jiang et al. (2023) examines a new approach to detecting fake news, which employs multimodal learning using query prompts. Traditionally, fake news detection was performed through the analysis of textual information, but this approach often overlooks the complexity and nuances of online disinformation. The new method proposed in the article includes the use of multimodal data (text and images) and a training methodology using query prompts, which allows for better detection of fake news, especially in situations with limited data. The authors utilize three types of query prompts with a soft verbalizer and a merging method that considers similarity for adaptively combining multimodal representations. This approach shows higher F1 scores and accuracy on two multimodal standard datasets, confirming its effectiveness in real-world scenarios.

The article by Syed et al. (2023) proposes a hybrid approach that combines weakly supervised learning and deep learning for detecting fake news on social networks. The study focuses on using machine learning methods, such as SVM, for annotating large volumes of unlabeled data, as well as applying deep learning techniques like Bi-LSTM and Bi-GRU for classifying fake news. The authors utilize TF-IDF and Count Vectorizer for feature extraction from textual data. Experimental results show that the proposed approach achieves high accuracy in detecting fake news, making it an effective tool in combating online disinformation.

The article by Xie and Li (2023) introduces the concept of “gatekeepers” in social networks for detecting fake news. Gatekeepers are active users who participate in the dissemination of news. The research proposes a gatekeeper behavior model based on Recurrent Neural Network (RNN), which includes training the model and detecting fake news. The method is capable of detecting fake news in real-time using data from Twitter and Weibo. Experimental results demonstrate that the Gated Recurrent Unit (GRU) achieves the best overall performance. The proposed method surpasses several contemporary approaches, showcasing its effectiveness in the early and middle stages of news dissemination.

In the article by Přibáň et al. (2019), the task of auto-mating the detection of fake news and fact-checking for West Slavic languages, specifically Czech, Polish, and Slovak, is addressed. The authors present datasets for these languages and conduct preliminary experiments that establish a baseline for further research in this area. They utilize 10-fold cross-validation to evaluate both balanced and unbalanced datasets, as well as conduct binary experiments with just the “TRUE” and “FALSE” classes. The input data for the classifier consists of either the statement text alone or the statement text supplemented by justification text.

In the article by Bucos and Drăgulescu (2023), the efficiency of using the back-translation (BT) method with transformer models to improve the detection of fake news in Romanian is explored. The study is based on data from Factual.ro, where models with BT showed better results in terms of accuracy, precision, recall, F1-score, and AUC compared to models trained on the original dataset. The use of mBART for BT with French as the target language improved the model’s performance compared to Google Translate. The Extra Trees Classifier and the Random Forest Classifier were among the best-performing models tested. The results indicate the potential of using BT with transformer models like mBART to enhance the effectiveness of fake news detection.

In the article by Afanasieva et al. (2022), the problem of determining the veracity of information, especially in the context of social unrest and significant events such as the US presidential elections and Russia’s invasion of Ukraine, is investigated. The authors examine the efficiency of using neural networks to detect fake news. To improve classification accuracy, a data preprocessing algorithm based on the fundamental principles of natural language processing was developed. The study identified linguistic patterns of fake news, which became the basis for data preprocessing. The features of convolutional and recurrent neural networks and their modifications for analyzing textual data are described. A set of metrics was chosen to compare certain models, characterizing the efficiency of algorithms. The accuracy of these models was tested on data related to the US presidential elections and the large-scale invasion of the russian into Ukraine.

Supplementary Table 1 presents a comparative analysis of different studies in the field of fake news detection using various datasets, models, languages of datasets, and approaches.

Based on the analysis of contemporary research in the field of disinformation detection presented in Supplementary Table 1, it can be concluded that existing approaches significantly focus on the English and Chinese languages, leaving the Ukrainian language with an insufficient level of attention. This indicates a lack of specialized datasets and models that would be adapted to the peculiarities of the Ukrainian language. In this context, the approach “Online Learning with Sliding Windows for Text Classifier Ensembles” (OLTW-TEC) has been developed, aimed at creating a comprehensive method for detecting disinformation in Ukrainian-language content. The method includes stages of data collection and preprocessing, analysis of sentiment, emotions, and text vectorization, allowing for a deeper analysis and more effective detection of fake news, relying on the unique linguistic and cultural features of the Ukrainian language.

## 3 Meta-model—OLTW-TEC

Let us introduce the enhancement of an ensemble authors Bodyanskiy et al. (2024) of adaptive predictors for multidimensional non-stationary sequences and its online training, aimed at improving efficiency in the context of text classification. This is achieved by integrating advanced natural language processing methods and adaptive learning algorithms. Specifically, vector characteristics of text documents, optimally selected for the given task, are used as input data for the classification model ensemble. The optimization of these models’ weights in the ensemble is performed using the Adam algorithm. An important aspect of the enhancement is the implementation of the “sliding window” method, which ensures the adaptability of the predictors to changes in the data. Such an approach allows for high accuracy and adaptability of models under the dynamic conditions of online learning.

Let us consider an ensemble of models for text classification $MP_{1},\ldots ,MP_{j},\ldots ,MP_{h}$ , each processing the vector characteristics of a text document obtained using the best model of vectorization. These characteristics are represented as $x\left(\tau \right)={\left(x_{1}\left(\tau \right),\ldots ,x_{i}\left(\tau \right)\right)}^{T}$ for τ=1,2,…,T . The estimate that appears at the output of each member of the ensemble will be denoted as $x\;{\left(\hat{\tau }\right)}_{j}$ for $h_{j}=1,2,\ldots ,h$ , where each $x\;{\left(\hat{\tau }\right)}_{j}$ represents the class score for the corresponding document. The members of the ensemble can include both traditional text classification models such as logistic regression or SVM, as well as more complex neural networks, including recurrent networks and deep learning structures like LSTM or transformers.

The estimates $x\;{\hat{\left(\tau \right)}}_{j}$ from each model of the ensemble are fed into the meta-model, which forms a combined prediction for text classification. The prediction of the meta-model is presented in the form


$$x^{*}\left(\tau \right)=\overset{h}{\underset{j=1}{\sum }}c_{j}x\;{\left(\hat{\tau }\right)}_{j}=x\;\left(\hat{\tau }\right)c\text{,}$$

where $c={\left(c_{1},\ldots ,c_{j},\ldots ,c_{h}\right)}^{T}$ is a weight vector that determines the contribution of each individual model in the ensemble.

The matrix $x\;\left(\hat{\tau }\right)=\left(x\;{\left(\hat{\tau }\right)}_{1},\ldots ,x\;{\left(\hat{\tau }\right)}_{j},\ldots ,x\;{\left(\hat{\tau }\right)}_{h}\right)$ is formed from the class estimates generated by each model. The parameters of the meta-model satisfy the condition of unbiasedness:


$$\overset{h}{\underset{j=1}{\sum }}c_{j}=c^{T}E_{h}=1\text{,}$$

where $E_{h}$ is a vector formed by ones. The weights c are adjusted in such a way as to optimize the overall accuracy of the meta-model’s classification.

To determine the optimal parameters of the meta-model (weight vector c), the Adam optimization algorithm is used, an alternative to traditional methods such as the Lagrange multipliers. The loss function $L\left(c\right)$ , which is minimized, is defined as the sum of the squares of the difference between the true classes and the predictions of the meta-model:


$$L\left(c\right)=\overset{T}{\underset{\tau =1}{\sum }}∥x\left(\tau \right)-x\;\left(\hat{\tau }\right)c{∥}^{2}\text{,}$$

where $x\left(\tau \right)$ represents the true class labels. Minimizing the loss function $L\left(c\right)$ using Adam allows for the efficient adjustment of the weights c, ensuring the optimal combination of predictions from different ensemble models to achieve high classification accuracy.

In cases of time-varying or non-stationary textual data, the efficiency of the meta-model can be enhanced using the “sliding window” method. This method involves updating the parameters of the meta-model using only the most recent s observations (text documents) from $x\left(T-s+1\right)to\;x\left(T\right)$ . When a new observation $x\left(T+1\right)$ arrives, the oldest observation in the “window” is removed, and the assessment is based on data from $x\left(T-s+2\right)to\;x\left(T+1\right)$ . This approach allows the meta-model to be more adaptable to changes in data patterns and improves its predictive ability based on current information, especially in situations where textual data is characterized by high dynamics or non-stationarity.

Choosing the optimal size of the “sliding window” s is crucial for achieving the highest classification accuracy in the ensemble of meta-models. This choice often relies on empirical considerations, as *a priori* knowledge about the nature of changes in textual data can be limited. In scenarios where different “sliding window” sizes may be optimal for different types of textual data, an effective approach is to create a set of meta-models, each built for a specific window size.

To determine the best meta-model, a second-level meta-model can be applied, which evaluates and selects the most efficient meta-model based on its performance across the entire training sample. This approach allows for dynamic adaptation to changes in textual data and selecting the best classification method depending on the specific context.

## 4 Method

The developed comprehensive method for detecting disinformation covers everything from data collection and preprocessing to sentiment analysis, emotion analysis, and text vectorization. This method includes the application of advanced machine learning techniques and neural networks, providing a deep analysis of textual data and effective detection of fake news. Supplementary Figure 1 illustrates the structure of the proposed method as a series of the following steps:

### 4.1 Step 1: data collection (block 1)

Data collection is a critically important stage in the process of developing a method for detecting disinformation. Properly collected and structured data allow for the effective training of machine learning models and analytics. Here is a more detailed description of this step:

#### 4.1.1 Collection of textual data

1. Source identification: determining sources for data collection is an important task. Sources can include news portals, social networks, blogs, forums, and other platforms where users can publish or share information.
2. Data collection automation (Gramyak et al., 2022): developing scripts or using existing tools for automatic data collection. This can include web scraping, social network API requests, and more.
3. Data filtering and validation: filtering and validating data to ensure their quality and relevance to research requirements.

#### 4.1.2 Metadata collection

1. Author information: collecting data about the authors of texts may include information about their profiles, publication history, number of followers, and other social indicators that can be useful for analysis.
2. Source information:. collecting information about the sources of texts, such as URL, publication date, number of views, likes, comments, and other indicators that can be important for analyzing the context and popularity of publications.
3. Structuring metadata. organizing collected metadata into structured databases for easy access and analysis in later stages of research.

### 4.2 Step 2: data preprocessing (block 2)

Data preprocessing is a fundamental step in the process of analyzing textual information. This step involves various techniques and methods that help prepare the collected data for further analysis. Here is a more de-tailed description of this step (Lipianina-Honcharenko et al., 2022a; Gramyak et al., 2022):

1. Tokenization (Lipianina-Honcharenko et al., 2023): the process of breaking text into individual words, phrases, symbols, or other meaningful elements called tokens. This assists in further analysis and processing of the text.
2. Stemming (Lipyanina et al., 2020): the process of removing suffixes, prefixes, and infixes from words to return them to their base form. This facilitates the detection of common themes and patterns in the text.
3. Part-of-speech tagging: the process of identifying the parts of speech of each word in the text, which can be useful for syntactic analysis and determining semantic relationships between words.
4. Named entity recognition: identifying and classifying named entities in the text, such as names of people, organizations, locations, etc.

### 4.3 Step 3: text vectorization (block 3)

Text vectorization is a crucial step that trans-forms textual data into a numerical format, convenient for analysis and processing using machine learning methods (Lipyanina et al., 2020). Let us look more closely at this process:

#### 4.3.1 Choosing the vectorization method

1. Word2Vec (Golovko et al., 2019): this model trains vector representations of words in a multidimensional space such that words that frequently occur together have similar vector representations.
2. GloVe:. another approach to word vectorization that uses both local and global statistical analysis of the text corpus to determine vector representations of words.
3. BERT: a modern model that uses attention mechanisms to determine relationships between words in text and can learn deep contextual representations of words.

#### 4.3.2 The vectorization process

1. Training or loading models: models can be trained on data or use pre-trained models for text vectorization.
2. Transforming text: applying the chosen model to transform each word in the text into vector representations.

#### 4.3.3 Building vector representations

1. Word vectorization: obtaining vector representations for each individual word in the text.
2. Text vectorization: aggregating vector representations of words to obtain vector representations of entire texts. This can be done through averaging, summing, or other aggregation methods.

### 4.4 Step 4: sentiment and emotion analysis (block 4)

Sentiment and emotion analysis is key to understanding the mood and nuances contained in textual data. This can help determine whether a text is positive, negative, or neutral, as well as identify potential emotional responses that may be associated with disinformation. Here’s a more detailed description of this step:

#### 4.4.1 Choosing models for sentiment and emotion analysis

Sentiment and emotion analysis, as outlined by Lipianina-Honcharenko et al. (2022), is crucial for understanding the mood and subtleties in textual data, particularly in identifying whether a text is positive, negative, or neutral, and recognizing emotional responses linked to disinformation.

1. Ready-made models: there are many pre-trained models for sentiment and emotion analysis, such as vader, textblob, or models based on bert and other deep neural networks.
2. Custom models: depending on the specific case, it may be useful to develop and train custom models on data specific to the task.

#### 4.4.2 Sentiment analysis

1. Calculating sentiment: applying models to determine the sentiment of each text, allowing to determine whether a text is positive, negative, or neutral.
2. Interpreting results: analyzing results to determine the overall mood of the data and identify possible anomalies or patterns.

#### 4.4.3 Emotion analysis

1. Calculating emotional tone: applying models to determine the emotional tone of the text, such as joy, sadness, anger, surprise, fear, etc.
2. Interpreting results: analyzing the obtained emotional tones to determine how emotions may be associated with disinformation and how they can be used for further analysis.

### 4.5 Step 5: online learning with sliding window for text classifier ensembles (block 5)

This step focuses on developing and training a classification model capable of detecting fake information based on text analysis and other identified features. Here’s a detailed description of this step:

#### 4.5.1 Creation and training of model ensemble

1. Creating an ensemble of models: selecting and creating various classification models (e.g., logistic regression, svm, lstm, transformers).
2. Training models: each model is trained separately on vectorized textual data.
3. Forming a meta-model: using an algorithm, such as adam, to optimize the weights of models in the ensemble, ensuring better integration and selection of predictions from each model.

#### 4.5.2 Implementing the “Sliding Window” method for online learning

1. Implementing “Sliding Window”: setting the size of the “sliding window” to select the most recent text documents that will be used for continuous updating and training of the models.
2. Online updating of models: periodically updating the ensemble models using the latest incoming data and discarding outdated data to maintain relevance and high prediction accuracy.
3. Adapting to changes in data: continuously adapting the meta-model to changes in textual data, ensuring an effective response to the dynamism and non-stationarity of text sequences.

### 4.6 Step 6: adaptation and retraining (block 8)

This stage aims to ensure the system’s ability to adapt to the evolution and change in forms of disinformation, guaranteeing its prolonged effectiveness in combating fake news (Golovko et al., 2019). Let us consider the details of this step:

#### 4.6.1 Retraining the system

1. Collecting new data: continuously gathering new data from open sources to reflect the latest trends and patterns of disinformation.
2. Assessing the need for retraining: analyzing the current efficiency of the system and determining if there is a need for retraining based on new data.
3. Retraining the model: applying the training process to the model using new data to adapt the model to new forms of disinformation.

#### 4.6.2 Updating models and algorithms

1. Analyzing new algorithms and technologies: evaluating and analyzing new algorithms and technologies that could be used to improve system efficiency.
2. Updating algorithms: making changes to the algorithms and methods used based on the information obtained and analysis of results.
3. Testing and validating updated models: conducting testing and validation of updated models to ensure their effectiveness and reliability.

#### 4.6.3 Monitoring and evaluation

1. Monitoring system efficiency: constantly monitoring the efficiency of the system to identify potential problems or areas for improvement.
2. Feedback and adaptation: collecting and analyzing feedback from users and experts for further improvement and adaptation of the system.

The comprehensive method for detecting disinformation outlined effectively integrates advanced machine learning techniques and neural networks, offering a highly adaptable and accurate tool for combating fake news through meticulous data collection, preprocessing, sentiment and emotion analysis, text vectorization, and the innovative application of online learning with sliding window techniques for text classifier ensembles.

## 5 Results

For the implementation of the proposed method, a dataset available on Kaggle (Ukrainian news, 2024) was chosen, which is a unique collection of approximately 60,000 news headlines collected from February 24 to December 11, 2022, covering the period of the full-scale russian-Ukrainian war. It includes both verified and fake news, collected from Ukrainian Telegram channels and russian channels with fakes, making it the largest open source of relevant data. The dataset contains two main attributes: the text of the news headline and a label indicating the veracity (True for confirmed news and False for fake news). In total, it contains 4,522 records with the label ‘False’. The data sources included Telegram channels such as “SUSPILNE NEWS,” “Perepichka NEWS,” and others.

To ensure the accuracy and completeness of the collected data, the process of data collection is carefully structured. The dataset was curated using automated scripts for data scraping and API requests to gather news headlines from Ukrainian and Russian Telegram channels, which are known to be primary sources of both verified and fake news during the russian-Ukrainian war. Specific criteria were established to select reliable sources, such as official news outlets like “SUSPILNE NEWS” and well-known channels that have been identified as disseminators of disinformation, like “Perepichka NEWS.” The selection process also involved filtering out irrelevant content to maintain the dataset’s focus on news items directly related to the conflict.

In the implementation phase, data was initially prepared, where textual materials were converted into a numerical format using the TF-IDF vectorization method. Subsequently, several individual models were trained based on these data, including logistic regression, SVM, random forest, gradient boosting, KNN, decision tree, XGBoost, and AdaBoost. Each model was adapted and optimized to solve the classification task using the training dataset. In the second phase of implementation, a meta-model based on XGBoost was formed, which was trained using predictions obtained from individual models through a stacking mechanism. The classification accuracy was evaluated on a test dataset, with results presented through a classification report and confusion matrix, visualized as a heatmap. This approach demonstrates the high potential of ensemble methods and stacking to enhance machine learning efficiency in complex text classification tasks, particularly in detecting disinformation.

The obtained classification results are presented in a report format (Supplementary Figure 2), which includes metrics such as precision, recall, f1-score, and support for two classes: “False” (fake news) and “True” (true news).

In the process of evaluating the effectiveness of the news classification model (Supplementary Figure 2A), an analysis was conducted on its ability to differentiate factual truths from fake messages. According to the results, the model shows a high overall classification accuracy of 93%. For the “False” class, which is presumed to identify fake news, the precision was 0.95, indicating a high probability of correctly classifying a news item as fake when the model does so. Meanwhile, the recall for this class is 0.72, indicating that about 28% of fake news was missed by the model. On the other hand, for the “True” class, corresponding to true news, the model showed an impressive recall level of 0.99, meaning it successfully identified 99% of true news. Such an ability of the model to effectively classify true news is an important feature in the context of combating disinformation.

The analytical approach to evaluating the model’s performance also included an analysis of the confusion matrix. In this matrix (Supplementary Figure 2B), 1,793 true negative results were noted, confirming the correct classification of fake news. However, 705 false negative results were identified, indicating the existence of a certain number of fake news that the model mistakenly classified as true. For the true news class, 8,138 true positive results and 99 false positives were registered, demonstrating high accuracy in detecting factually true content. The F1-score for both classes, which harmonizes precision and recall, was found to be 0.82 for “False” and 0.95 for “True,” overall confirming the model’s balance and reliability.

Analyzing the results presented in Supplementary Table 2, scientifically substantiated conclusions can be made about the effectiveness of the OLTW-TEC method compared to classic classification approaches. Examining each metric individually, it is evident that the OLTW-TEC method demonstrates significant improvements across most parameters.

In the context of precision for the “False” class, OLTW-TEC achieves a value of 0.95, which is comparable to logistic regression and surpasses other classic methods, except for Gradient Boosting. This indicates OLTW-TEC’s high ability to correctly identify fake news. Regarding the “True” class, OLTW-TEC’s precision is 0.92, a competitive figure, especially compared to SVM and XGBoost.

The recall rate for the “False” class in OLTW-TEC at 0.72 is significantly higher than that of logistic regression and matches the performance of SVM, indicating the model’s improved ability to detect fake news among the negative class. For the “True” class, the recall rate is 0.99, a common trend among all considered models, highlighting their capability to identify true news.

The harmonic mean between precision and recall, defined as the F1-score, for OLTW-TEC is 0.82 for the “False” class and 0.95 for the “True” class, effectively confirming the model’s balance between these two crucial metrics. This significantly exceeds the F1-scores of logistic regression for the “False” class and is on par with the best results among other classic methods.

The overall accuracy of OLTW-TEC is 93%, which is one of the highest figures among all the models considered (Supplementary Table 2), confirming its strong position as a reliable tool for news classification. The confusion matrix also indicates a high number of correctly classified instances for both classes.

## 6 Discussion

Analyzing the results presented in Supplementary Table 1, significant achievements in the field of fake news detection using various approaches, ranging from deep learning to multi-modal analysis, were identified. However, an important gap in existing research was discovered, namely the insufficient attention to Ukrainian-language content. Our OLTW-TEC method, focused on a Ukrainian dataset, fills this gap, highlighting the importance of developing specialized solutions for specific linguistic and cultural contexts.

Comparative analysis with other studies (see Supplementary Table 1) showed that OLTW-TEC achieves significant results in accuracy, demonstrating values of 0.95 for the “False” class and 0.92 for the “True” class. These metrics indicate the method’s high efficiency in detecting fake news and true messages, respectively. Particularly valuable is OLTW-TEC’s ability to provide a high recall (recall) for the “False” class at 0.72, higher compared to some other methods, such as logistic regression. This indicates the method’s effectiveness in the context of detecting fake news, which often have complex patterns and can be disguised as credible.

F1-scores, harmonizing precision and recall, also highlight OLTW-TEC among other studies, underscoring its balance and reliability. An overall accuracy of 93% demonstrates that OLTW-TEC can serve as a reliable tool in the fight against disinformation and in detecting fake news. The confusion matrix confirms the model’s high accuracy with a minimal number of false classifications, which is especially important in situations where ensuring information accuracy is crucial to prevent panic or disinformation during critical moments.

It’s worth noting that although OLTW-TEC shows significant results compared to classic methods, there are certain limitations that should be considered when interpreting these results. Firstly, the use of ensemble methods and the “sliding window” technique requires substantial computational resources, which may limit the widespread application of OLTW-TEC in real-time, especially on devices with limited computing power. This raises concerns about the scalability of the method, particularly when applied to large-scale datasets or deployed in environments with constrained computational resources.

Secondly, the method may be sensitive to the size of the “sliding window,” as an incorrect choice of size can lead to overfitting or underfitting of the model, especially in conditions where data dynamics change rapidly. This requires further research to optimize parameters and improve the model’s adaptability, ensuring that the method can maintain high accuracy across different contexts and data flows.

Thirdly, although OLTW-TEC demonstrated high efficiency on Ukrainian-language data, its universality and efficiency in datasets of other languages and cultural contexts have not been fully explored. Further experiments are needed to determine whether the method can maintain similar performance metrics in other linguistic environments. This would involve testing the model on multilingual datasets and assessing its robustness across diverse linguistic and cultural contexts.

Finally, considering the rapid development of technology and the changing nature of disinformation dissemination, there is a need for continuous updating and adaptation of the model to maintain its relevance. This concerns not only algorithmic improvements but also the collection and integration of new data for training, which in turn requires additional efforts to ensure the reliability and quality of the input data.

Given these considerations, it would be beneficial to address the scalability and computational efficiency of OLTW-TEC in future research. This could involve exploring methods to reduce computational load, such as model pruning, optimizing algorithmic complexity, or employing more efficient hardware solutions. Additionally, incorporating distributed computing techniques or leveraging cloud-based platforms could help manage the computational demands of the method, making it more accessible for broader applications.

Overall, while OLTW-TEC shows promising results, it is important to emphasize the need for continued development and adaptation of the method to effectively respond to the constantly changing challenges in the field of disinformation detection. Future work should focus on addressing the identified limitations to enhance the scalability, efficiency, and applicability of the proposed approach across different contexts and environments.

Future research will focus on enhancing the OLTW-TEC method, particularly on expanding its computational efficiency and adaptability. One of the priorities is the optimization of algorithms to reduce computational power requirements, allowing the method to be applied in a wider range of applications, including mobile and other de-vices with limited resources. Attention will also be focused on more precise tuning of the “sliding window” parameters to improve prediction accuracy in various conditions of dynamic data flow. Another important direction is the expansion of method valida-tion on diverse linguistic datasets, which will determine its universality and effective-ness in a global context. Additionally, mechanisms for continuous updating of the training dataset are planned to be implemented, allowing the system to adapt to new patterns and forms of disinformation. These measures aim not only to improve the accuracy and reliability of OLTW-TEC but also to ensure its resilience to rapid changes in the digital information space.

## 7 Conclusion

In the course of the conducted research, the effectiveness of the OLTW-TEC method was thoroughly examined and evaluated. This innovative approach addresses the challenge of text classification in the context of non-stationary data and dynamic online learning. The methodology incorporates the use of an ensemble of adaptive classifiers, vector representations of texts, optimization of classifier weights using the Adam algorithm, and the implementation of a “sliding window” mechanism to maintain the model’s relevance.

According to the evaluation results presented in the classification report, OLTW-TEC demonstrated high accuracy, achieving a 93% performance rate. Precision and recall for the “False” class were found to be 0.95 and 0.72, respectively, indicating high efficiency in detecting fake news with a minimal percentage of false negatives. For the “True” class, the model provided almost perfect recall with a rate of 0.99, thereby confirming its ability to recognize true news. The obtained results illustrate that OLTW-TEC possesses a high balance between different metrics, ensuring reliable and effective classification in real-world conditions.

The analysis of the confusion matrix points to a high degree of classification accuracy, with a minimal number of false positives and false negatives. This demonstrates the reliability of OLTW-TEC as a tool for information filtering, which is particularly relevant in the context of combating disinformation.

Compared to traditional classification methods (see Supplementary Table 1), OLTW-TEC not only shows better results across most metrics but also provides room for adaptation to changes in the nature of the data. The choice of “sliding window” size and the possibility of adjusting it depending on the data specifics give the method additional flexibility and precision.

## Data Availability

The original contributions presented in the study are included in the article/Supplementary material, further inquiries can be directed to the corresponding author.
