# Supplementary material for: OLTW-TEC: online learning with sliding windows for text classifier ensembles
Source: Front Artif Intell. 2024 Sep 11;7:1401126. doi: 10.3389/frai.2024.1401126 (PMC11422347; doi:10.3389/frai.2024.1401126)
Supplement: Supplementary file 1 [file Data_Sheet_1.docx]

Supplementary Material

# Supplementary Figures and Tables

## Supplementary Figures


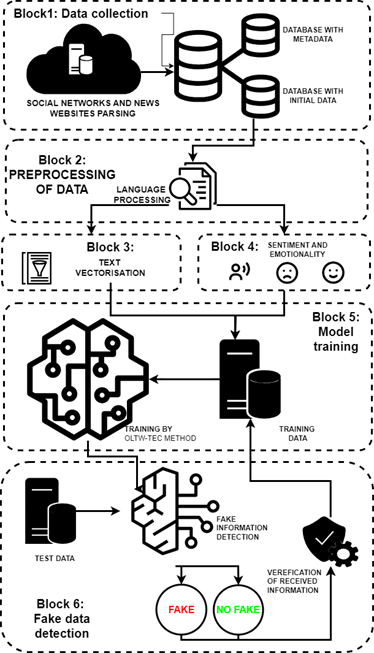


**Supplementary Figure 1.** Structure of the intelligent method for online disinformation detection

| 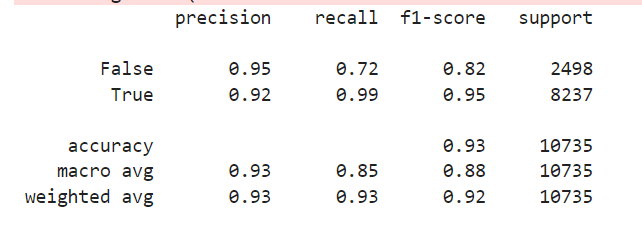 | 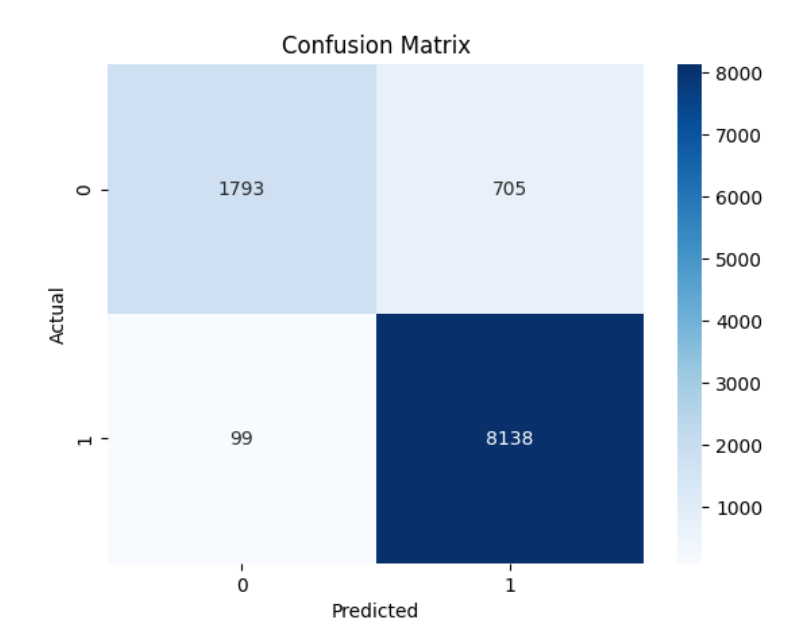 |
| --- | --- |
| A | B |

**Supplementary Figure 2.** Evaluation results

# Supplementary Tables

Table 1. Comparison of similar studies.

| **Authors** | **Dateset** | **Model** | **Accuracy (Acc.)** | **Labguage** | **Approach** |
| --- | --- | --- | --- | --- | --- |
| **Chang, Q., Li, X., Duan, Z.[11]** | Politifact | GCN-GANM | 0.8602 | English | Deep Learning |
|  | Gossipcop |  | 0.9825 | English |  |
|  | Weibo |  | 0.9804 | Chinese |  |
| **Peng, L., Jian, S., Kan, Z., Qiao, L., Li, D.[12]** | Weibo | CSFND | 0.895 | Chinese | Contextual Semantic Representation |
|  | Twitter |  | 0.833 | English |  |
| **Ahammad, T.[13]** | COVID-19 News Headlines | LDA, VADER | Not specified | English | Sentiment Analysis and Topic Modeling |
| **Qu, Z., Meng, Y., Muhammad, G., Tiwari, P.[14]** | Gossip | QMFND | 0.879 | English | Quantum Multimodal Fusion |
|  | Politifact |  | 0.846 |  |  |
| **Farhangian, F., Cruz, R. M. O., Cavalcanti, G. D. C.[15]** | Liar | Різні (включаючи SVM, LR, KNN, NB, RF, AdaBoost, XGBoost, MLP, CNN, BiLSTM, трансформерні моделі) | SVM з LLaMA - 0.266 | English | Comprehensive Analysis and Comparison of Various Approaches to Fake News Detection |
|  | ISOT |  | This is achieved by BiLSTM or CNN with methods such as BERT, DistilBERT, BART, RoBERTa, LLaMA, and Falcon, reaching up to 100% F1-score. |  |  |
|  | COVID |  | BiLSTM з Falcon - 95% |  |  |
|  | GM |  | BiLSTM з Falcon - 99.3% |  |  |
| **Fang, X., Wu, H., Jing, J., Meng, Y., Yu, B., Yu, H., & Zhang, H.[16]** | Chinese | NSEP | 86.8% Chinese in dataset | Chinese | Detection of Fake News through Perception of News Semantic Environment (NSEP) |
| **Soga, K., Yoshida, S., & Muneyasu, M.[17]** | Custom (Twitter), | Graph Transformers | 86.41% на Custom, | English | Detection of Fake News Using Positional Similarity and Graph Neural Networks |
|  | FibVID |  | 79.80% на FibVID |  |  |
| **Yang, H., Zhang, J., Zhang, L., Cheng, X., & Hu, Z.[18]** | Weibo, | MRAN | 90.3% на Weibo, | Chinese, | Multimodal Analysis with Consideration of Interconnections |
|  | Twitter, |  | 85.5% на Twitter, | English |  |
|  | Pheme |  | 87.0% на Pheme | English |  |
| **Raja, E., Soni, B., Lalrempuii, C., & Borgohain, S. K.[19]** | Dravidian_Fake | Hybrid model DTCN_BiLSTM_CAM | 93.97% | Dravidian languages | Hybrid Approach with DTCN, BiLSTM, CAM |
| **Luvembe, A. M., Li, W., Li, S., Liu, F., & Wu, X.[20]** | GossipCO, | CAF-ODNN | GossipCO Dataset: 86.3% | English | Multimodal Approach with Complementary Attention and Optimized Deep Neural Network |
|  | PolitiFact, |  | PolitiFact Dataset: 88.9% |  |  |
|  | Fakeddit, |  | Fakeddit Dataset: 90.0% |  |  |
|  | Pheme |  | Pheme Dataset:: 87.9% |  |  |
| **Jiang, Y., Yu, X., Wang, Y., Xu, X., Song, X., & Maynard, D.[21]** | PolitiFact | SAMPLE (D-SAMPLE, C-SAMPLE, M-SAMPLE) | F1/Acc: 0.81/0.82 (PolitiFact, M-SAMPLE, Data rich) | English | Multimodal Learning Using Query Templates |
|  | GossipCop |  | F1/Acc: 0.58/0.62 (GossipCop, M-SAMPLE, Data rich) |  |  |
| **Syed, L., Alsaeedi, A., Alhuri, L. A., & Aljohani, H. R.[22]** | Twitter (195,943 tweets) | Bi-LSTM, Bi-GRU, SVM (Weakly Supervised) | Bi-LSTM: 89.95, Bi-GRU: 89.60 | English | Hybrid Approach with Weakly Supervised Learning and Deep Learning |
| **Xie, B., & Li, Q.[23]** | Twitter, Weibo | RNN-Based Gatekeepers' Behavioral Model (RGBM) | Accuracy: 98.5, Відгук: 97.8, F1: 97.6, Loss: 0.058 | English, Chinese | Detection of Fake News Using Gatekeepers' Behavioral Model |
| **Přibáň, P., Hercig, T., & Steinberger, J.[24]** | Czech | Logistic regression | Unbalanced (2 classes): Accuracy - 0.35 / 0.82 | Czech, | Machine Learning, 10-Fold Cross-Validation |
|  | Polish language |  | Unbalanced (2 classes): Accuracy - 0.34 / 0.73 | Polish, |  |
|  | Czech |  | Unbalanced (2 classes): Accuracy - 0.35 / 0.82 | Czech, |  |
| **Bucos, M., & Drăgulescu, B.[25]** | Factual.ro | Extra Trees Classifier, Random Forest Classifier, Support Vector Machine, Logistic Regression | Acc: 0. 0.8075, Pre: 0.8548, Rec: 0.8217, F1: 0.8084, AUC: 0.8451, Kappa: 0.6012 | Romanian | Using Transformer Models for Back Translation (BT), including mBART and Google Translate, to Improve Fake News Detection. |
| **Afanasieva, I., Golian, N., Golian, V., Khovrat, A., & Onyshchenko, K.[26]** | Data on the U.S. presidential elections and Russia's invasion into Ukraine | CNN, RNN | CNN: 93-94% (war), 94% (elections); RNN: 94% (war), 96% (elections) | English, russian | Using Neural Networks for Fake News Detection |

**Table 2.** Comparison with the Developed Method

| **Model** | **Precision (False)** | **Precision (True)** | **Recall (False)** | **Recall (True)** | **F1-score (False)** | **F1-score (True)** | **Accuracy** | **Confusion Matrix (False, True)** |
| --- | --- | --- | --- | --- | --- | --- | --- | --- |
| Logistic Regression | 0.96 | 0.85 | 0.44 | 0.99 | 0.60 | 0.92 | 86.42% | (1091, 1407), (51, 8186) |
| SVM | 0.90 | 0.93 | 0.75 | 0.99 | 0.85 | 0.96 | 90.61% | (1680, 818), (68, 8169) |
| Random Forest | 0.88 | 0.95 | 0.81 | 1.00 | 0.89 | 0.97 | 90.31% | (2026, 472), (31, 8206) |
| Gradient Boosting | 0.94 | 0.86 | 0.48 | 0.99 | 0.64 | 0.92 | 87.31% | (1209, 1289), (73, 8164) |
| KNN | 0.91 | 0.87 | 0.51 | 0.99 | 0.66 | 0.92 | 87.57% | (1285, 1213), (121, 8116) |
| Decision Tree | 0.81 | 0.95 | 0.83 | 0.94 | 0.82 | 0.94 | 91.45% | (2077, 421), (497, 7740) |
| XGBoost | 0.89 | 0.89 | 0.61 | 0.98 | 0.73 | 0.93 | 89.19% | (1533, 965), (195, 8042) |
| AdaBoost | 0.88 | 0.87 | 0.50 | 0.98 | 0.64 | 0.92 | 86.92% | (1259, 1239), (165, 8072) |
| OLTW-TEC | 0.95 | 0.92 | 0.72 | 0.99 | 0.82 | 0.95 | 93% | (1793, 705), (99, 8138) |
